# Klotho Null Mutation Indirectly Leads to Age-Related Lacrimal Gland Degeneration in Mutant Mice

**DOI:** 10.3390/biology12101328

**Published:** 2023-10-11

**Authors:** Chun-Yen Wu, Da-Fong Song, Tsung-Han Lu, Zhi-Jia Chen, Su-Min Tsai, Ya-Jing Liu, Han-Hsin Chang, David Pei-Cheng Lin

**Affiliations:** 1 Department of Nutrition, Chung Shan Medical University, Taichung City 402, Taiwan; 2 Department of Medical Laboratory and Biotechnology, Chung Shan Medical University, Taichung City 402, Taiwan; 3 Department of Ophthalmology, Chung Shan Medical University Hospital, Taichung City 402, Taiwan

**Keywords:** Klotho mutation, lacrimal gland degeneration, tear volume, acinar atrophy, age-related dry eye disease, murine model

## Abstract

**Simple Summary:**

The Klotho gene null mutation has been shown to cause accelerated senescence in many organs. Nevertheless, whether the mutation may cause lacrimal gland dysfunction and degeneration has not been elucidated. This study shows that the Klotho null mutation leads to reduced tear volume with characteristics of lacrimal gland degeneration, including glandular and acinar atrophy, thickened capsules, and collagen deposition. The mechanism analysis showed a multi-fold pathogenesis process, including chronic elevated oxidative stress, constant extracellular matrix remodeling, and epithelial−mesenchymal transition. The results support the concept that Klotho null mutant mice may be a study model for age-related dry eye disease.

**Abstract:**

The Klotho null mutation is known to lead to accelerated aging in many organs, but its effects on tear secretion and lacrimal gland (LG) senescence have not been addressed. This study investigated whether the Klotho null mutation would lead to a dry eye status and the outcome of LG without Klotho function. The Klotho (^−/−^) mutant mice showed reduced LG size and tear volume on the 8th week, as compared to their littermates (^+/+, +/−^). Hematoxylin–Eosin and Masson’s trichrome staining were performed to determine morphological changes and collagen deposition. Traits of LG aging, including acinar atrophy, thickened capsules, and more collagen depositions, were observed. Immunohistochemical detections for Klotho, α-SMA, MDA, 8-OHdG, vasoactive intestinal polypeptide (VIP), tyrosine hydroxylase (TH), MMP-2, MMP-9, and FGF-23 were performed and compared among the three genotypes (^+/+^, ^+/−^, ^−/−^) at 6 and 8 weeks of age for mechanism analyses. Unexpectedly, the Klotho protein was not detected in the LG of all the three genotypes, indicating indirect effects from the Klotho null mutation. Further analyses showed abundant MDA and 8-OHdG detected in the Klotho (^−/−^) LG on the 8th week, indicating elevated oxidative stress. In addition, both sympathetic and parasympathetic neural transducing activities, as represented by TH and VIP expression, respectively, and α-SMA were increased in LGs with Klotho mutations. Furthermore, MMP-2 and MMP-9 expression were elevated, with FGF-23 expression being decreased on the 8th week in the Klotho (^−/−^) LG. In conclusion, characteristics of age-related LG degeneration were found in the Klotho null mutant mice. These traits support the use of Klotho mutant mice as a model of age-related dry eye disease.

## 1. Introduction

Aging is highly related to increased susceptibility to diseases [1], and dry eye is among the commonest ocular diseases associated with age [1,2,3], as evidenced by many epidemiological studies [4]. Dry eye is a multifactorial disease of the tear and ocular surface, typically manifesting in eye discomfort, tear film instability, and reduced visual quality, and it can lead to apparent visual impairment in severe cases [1,4].

Lacrimal gland (LG) degeneration plays a pivotal role among all the pathological processes underlying dry eye status, as the LG is the major source of aqueous tear secretion. LG function decreases gradually with aging, as reflected by the reduction in tear secretion in the elderly [5,6]. Thus, LG degeneration over time is regarded as one of the main factors in age-related dry eye disease [7]. The characteristics of LG degeneration have been shown to include the increase in gland capsular thickness, atrophy, and fibrosis and diminished epithelial cell proliferation, leading to a dry eye status [7].

Although various histopathological changes that cause tear dysfunction have been reported in human LGs [8], it remains unclear how the changes cause dry eye disease (DED) chronically [7]. Therefore, further research is needed to understand the entire scenario, preferentially with animal models that develop dry eye disease in an age-dependent manner. Rodents are frequently used as study models for human dry eye research [3,6] based on their structural and functional similarities [9,10]. For example, normal C57BL/6 male mice would develop dry eyes and related ocular surface damage after one year of age [7]. However, research using a normal mouse model is tedious and therefore not the most convenient for mass screening candidates to prevent or slow down age-related changes.

The Klotho gene was identified as a suppressor gene against aging in mice, with disrupted Klotho gene function leading to accelerated senescence [11]. Conversely, the mouse life span was extended when Klotho was overexpressed [11]. The Klotho proteins exist in two forms: a membrane-bound and a secreted Klotho protein [11]. The membrane Klotho forms a complex with its co-receptor for FGF-23 (fibroblast growth factor-23) to facilitate phosphate excretion into urine [11]. With the Klotho null mutation, phosphates are accumulated within the body.

Although the Klotho null mutation has been shown to cause accelerated aging in many organs, its effects on the LG have not been elucidated. Because most homozygous Klotho null mutant mice would not survive beyond 10 weeks of age [11], they may be more suitable for investigating the entire scenario of age-related dry eye in a relatively short time. This study investigated whether the LG would exhibit accelerated aging characteristics with the Klotho null mutation to explore the possibility of using the mutant mice as a more applicable age-related dry eye disease model.

## 2. Materials and Methods

### 2.1. Klotho Mutant Mice and Genotyping

The Klotho mutant mice with genetic background on C57BL/6J were obtained from the Mutant Mouse Resource & Research Center (MRRC), UC Davis, USA. The strain was maintained in the National Laboratory Animal Center (NLAC), Taipei, Taiwan, and transferred to Chung Shan Medical University, Taichung City, Taiwan. The mice were housed in an animal facility maintained at 20 to 24 °C with 50% to 55% humidity under a 12 h light/12 h dark cycle and given a commercial diet and water ad libitum. All animal care and experimental procedures were performed following the standard laboratory animal protocols that had been approved by the Institutional Animal Care and Use Committee of Chung Shan Medical University (IACUC Approval No: 2472) in accordance with the ARVO Statement for the Use of Animals in Ophthalmic and Vision Research.

Only male mice were used in this study. The mouse genotypes were determined via PCR amplification based on a protocol developed by the Mutant Mouse Resource & Research Center (MMRRC) at the University of California, Davis. Two sets of primers were used as listed in Table 1.

The thermocycler parameters were set as 94 °C, 5 min for initiation; 94 °C, 15 s for denaturation; 65 to 55 °C 15 s for annealing; 72 °C, 30 s for elongation. The denaturation–annealing–elongation was processed for 40 cycles, followed by a final amplification at 72 °C for 5 min and the preservation of PCR products at 15 °C after the process finished.

### 2.2. Tear Volume Assessment

The mice were anesthetized via intraperitoneal injection with Avertin (2-2-2 tribromoethanol (Sigma-Aldrich, St. Louis, MO, USA, cat no. T48402, at 20 mg/mL). After the mice were completely anesthetized, a 1 mm width phenol red thread was inserted into the lower fornix for 20 s, followed by reading the wetted length. The average of triple repeats was obtained for each eye, allowing an interval of at least 30 min between tests.

### 2.3. Histological Preparation and Hematoxylin-Eosin Stain

The extraorbital lacrimal glands were removed from euthanized mice and fixed in 10% neutral formaldehyde for 18–24 h. The preparations were washed with phosphate-buffered saline and then subjected to increasing ethanol concentrations from 30%, 50%, 70%, 90%, and then 100% 3 times. Each ethanol treatment lasted for 8–12 h to ensure thorough dehydration. The samples were processed through xylenes and embedded in Surgipath Paraplast (Leica, Deer Park, IL, USA, cat no. 39601006). Tissue sections were cut at 5 μm thickness and dried at 60 °C. The sections were then processed for Hematoxylin–Eosin staining through dewaxation, rehydration, and staining in Mayer’s Hematoxylin (Agilent Technologies, Santa Clara, CA, USA, cat. no. S3309) for 90 s, followed by being washed 3 times and being de-stained with acidic alcohol for 10 s. After being stained with 3% Eosin Y (Sigma-Aldrich, St. Louis, MO, USA, cat. no. E6003), the slides were mounted by coverslips with Micromount (Leica, Deer Park, IL, USA, cat. no. 3801731).

### 2.4. Masson’s Trichrome Stain

A trichrome stain kit (Abcam, Cambridge, UK, cat. no. ab150686) was applied for staining. Firstly, Bouin’s fluid was pre-heated in a 60 °C oven and then applied onto de-waxed slides, covered with coverslips for 1 h also in a 60 °C oven, allowed to cool down under room temperature, and washed in ddH_2_O for 10 min. The preparations were then immersed in pre-mixed Weigert’s Iron Hematoxylin A and B for 5 min, washed in ddH_2_O for 2 min, and then, Biebrich Scarlet/Acid Fuchsin solution was applied for 15 min, followed by washing with ddH_2_O. After washing, phosphomolybdic/phosphotungstic acid was applied for 20 min, and aniline blue solution was applied for 15 min. The slides were then washed with ddH_2_O, covered with an acetic acid solution for 5 min, and then dehydrated in 95% ethanol for 2–3 s and in 100% ethanol for 2–3 s. The preparations were then air-dried and mounted by coverslips with Micromount (Leica cat. no. 3801730).

### 2.5. Immunohistochemistry

A Super Sensitive Polymer HRP IHC Detection System (BioGenex Laboratories, Fremont, CA, USA, cat. no. QD400-60KE) was used for immunohistochemical detection. Briefly, the slides were de-waxed, rehydrated, and micro-waved for 10 min 3 times in citrate buffer for antigen retrieval, followed by stepwise cooling down under running tap water. The preparations were then immersed in 5% H_2_O_2_ for 10 min, washed 3 times in tap water, and put into 1% blocking buffer for 10 min. A specific antibody was then applied onto the slides, and each slide was covered with a coverslip and kept under 4 °C overnight for antibody binding. The slides were immersed in TBS to release the coverslips and the sections were covered with super enhancer solution at room temperature for 40 min. The super enhancer solution was washed away with TBS and replaced by an HRP conjugate for 2 hrs. After thoroughly washing with TBS, DAB (3,3’-Diaminobenzidine) was applied for color detection and counterstained by Hematoxylin. The details of antibodies used for detection were Recombinant anti-Klotho antibody (Abcam, Cambridge, UK, cat. no. ab181373, dilution at 1/100), anti-α-SMA (alpha smooth muscle actin) (Abcam, Cambridge, UK, cat. no. ab7817, dilution at 1/500), anti-MDA (malondialdehyde) (Abcam, Cambridge, UK, cat. no. ab243066, dilution at 1/500, anti-8-OHdG (8-hydroxy-2-deoxyguanosine) (GeneTex, Irvine, CA, USA, cat. no. GTX35250, dilution at 1/400), anti-VIP (vasoactive intestinal polypeptide) (GeneTex, Irvine, CA, USA, cat no. GTX129461, dilution at 1/500), anti-TH (tyrosine hydroxylase) (GeneTex, Irvine, CA, USA, cat no. GTX113016, dilution at 1/500), MMP-2 (GeneTex, Irvine, CA, USA, cat. no. 104577, dilution at 1/500), MMP-9 (Abcam, Cambridge, UK, cat. no. ab38898, dilution at 1/300), and FGF-23 (MyBioSource, San Diego, CA, USA, cat. no. MBS2003657, dilution at 1/250).

DAB-positive signal quantification was performed using the ImageJ bundled with 64-bit Java 8 (National Institutes of Health, Bethesda, MD, USA) program with the Colour Deconvolution 2 plugin (a free software provided by Gabriel Landini) [12]. The procedure was conducted following a previously published protocol [13]. Briefly, the images were loaded and processed through color deconvolution, adding vectors, adjusting the threshold to exclude non-positive areas, and manually measuring the defined region of interest. The final results were expressed as the area of interest divided by the total area of acini.

### 2.6. Statistics

GraphPad Prism v9 (Boston, MA, USA) was used to perform two-way ANOVA (or mixed model) for assessment among wildtypes, Klotho heterozygotes, and Klotho homozygotes. All the quantification was presented as averages from triple repeats by two researchers who were blind to the study groups. The sample size indicated in each experiment was the number of mice. A significant difference was considered when the *p* value was less than 0.05. A highly significant difference was considered when the *p* value was less than 0.01 or 0.001.

## 3. Results

### 3.1. Decreased Tear Secretion and LG Size and Lack of Klotho Protein within LG

**Figure 1:**
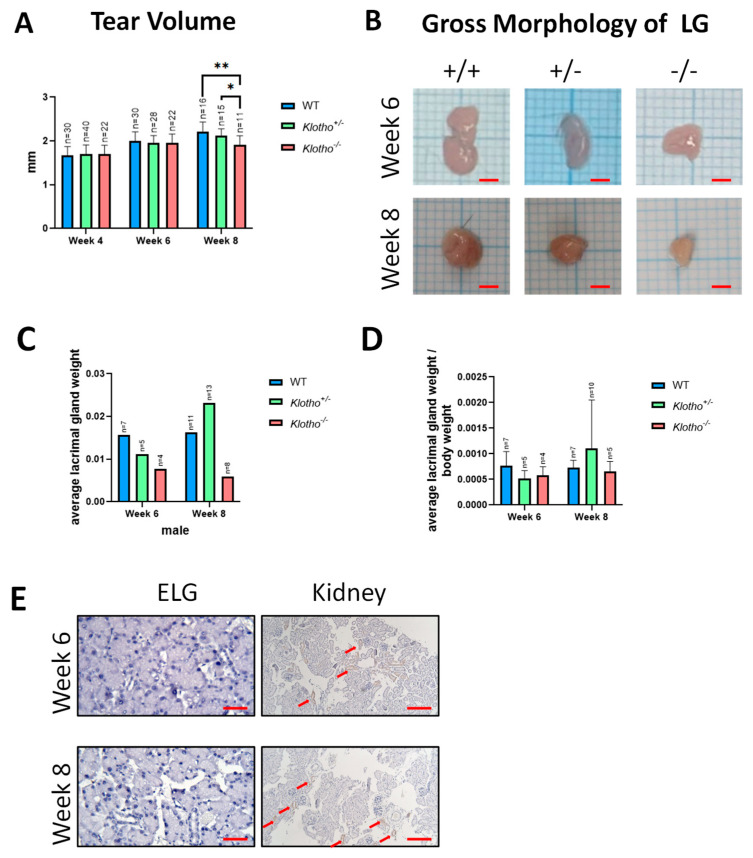
(**A**) Reduction in tear secretion in the Klotho null mutant mice. The tear volume, as reflected by the wetted length in millimeters (mm) on tear strips, was measured in the three mouse genotypes at 4, 6, and 8 weeks of age. At the 8th week of age, the Klotho^−/−^ mice showed significantly decreased tear volume compared to their Klotho^+/−^ and Klotho^−/−^ littermates, with * *p* < 0.05 and ** *p* < 0.01, respectively. (**B**) A representative photograph showed that the LG size of Klotho^−/−^ mice was reduced. (**C**) Quantification of average lacrimal gland weight. (**D**) Quantification of average lacrimal gland weight/body weight. (**E**) Immunohistochemical detection of the Klotho protein in the extraocular LG and kidney of the Klotho^+/+^ mice at 6 and 8 weeks of age. The kidney readily showed signals (indicated by red arrows) of the Klotho protein, unlike the lack of the Klotho protein in the LG. Scale bars represent 25 μm.

The tear volume of the Klotho^+/+^, Klotho^+/−^, and Klotho^−/−^ mice was assessed at 4, 6, and 8 weeks of age. As weeks of age increased, the tear volume also increased in the three genotypes. However, the Klotho^−/−^ mice had significantly less tear volume as compared to their Klotho^+/+^ and Klotho^+/−^ littermates (Figure 1A). The Klotho^−/−^ mice also showed reduced LG size (Figure 1B) and had less LG weight (Figure 1C), although without statistical significance. The reduction in LG size and weight was correlated with the general reduction in the body weight (Figure 1D) of the Klotho^−/−^ mice. To further understand the impact of the Klotho null mutation on the LG, we performed the immunohistochemical detection of the Klotho protein on the Klotho^+/+^ LG tissue sections. Unexpectedly, no Klotho protein was detected, unlike the readily detected signals in the kidney (Figure 1E).

### 3.2. Lacrimal Gland Atrophy and Capsular Thickening

**Figure 2:**
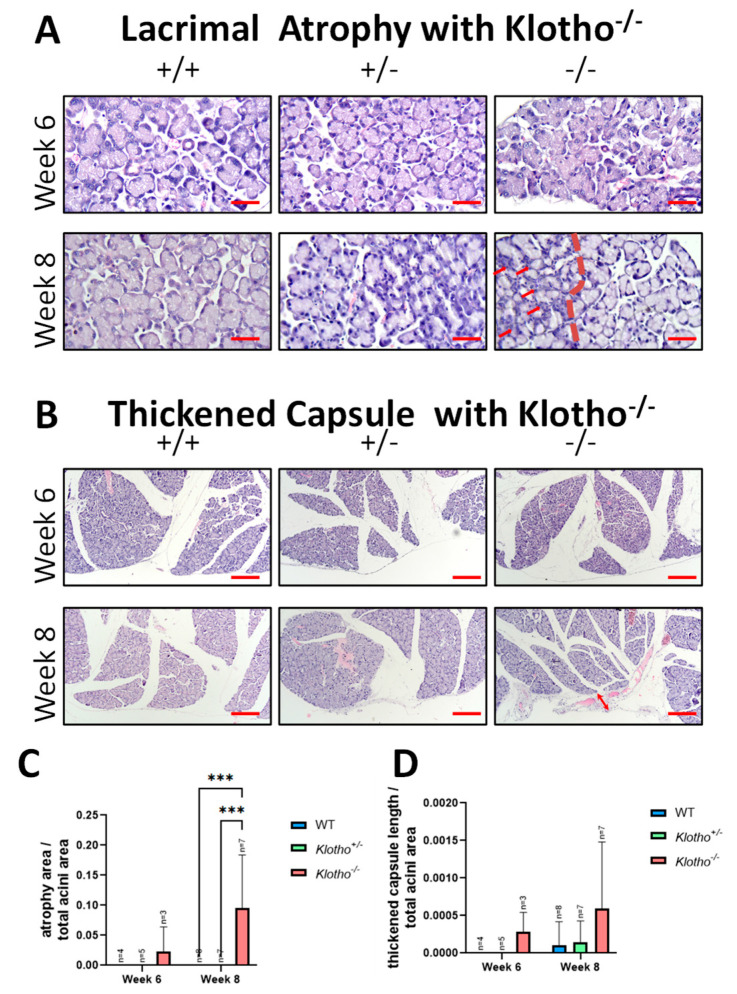
(**A**) Representative photographs of LG tissue sections of the three genotypes stained with Hematoxylin–Eosin. Acinar atrophy was evident on the left side (demarcated with a red dot line) of the Klotho^−/−^ LG at the 8th week of age. Scale bars represent 25 μm. (**B**) Thickening of the LG capsule (indicated by a red arrow) was found in the Klotho^−/−^ mice at 8 weeks of age. Scale bars represent 100 μm. (**C**) As presented by atrophy area/total acini area, the quantitative analysis of LG atrophy showed a significant difference with *** *p* < 0.001 when the Klotho^−/−^ LGs were compared with the Klotho^+/+^ or Klotho^+/−^ LGs. (**D**) Quantitative analysis of LG capsular thickening, as presented by thickened capsule length/total acini area, showed an increase in the Klotho^−/−^ LG, although without statistical significance.

To further characterize the changes in the LG, tissue sections of the three genotypes at the 6th and 8th week of age were stained with Hematoxylin–Eosin and compared. No significant difference among the three genotypes was observed in the 6th week (Figure 2A). In the 8th week, evident acinar atrophy (indicated by red arrows) was observed in the Klotho^−/−^ LGs, as exemplified by the left side of the Klotho^−/−^ LG shown in Figure 2A (demarcated by a red dot line). No comparative atrophy was observed in the Klotho^+/+^ and Klotho^+/−^ littermates at the same age. The quantitative analysis of the LG atrophy, as presented by atrophy area/total acini area, showed a significant difference (Figure 2C). In addition, LG capsular thickening appeared in the Klotho^−/−^ LG at the 8th week of age (Figure 2B, indicated by a red arrow), which was not observed in the LGs of their Klotho^+/+^ or Klotho^+/−^ littermates. The quantitative analysis of the LG capsular thickening, as presented by thickened capsule length/total acini area, showed an increase in the Klotho^−/−^ LG, although without statistical significance (Figure 2D).

### 3.3. Deposition of Collagen Fibers

**Figure 3:**
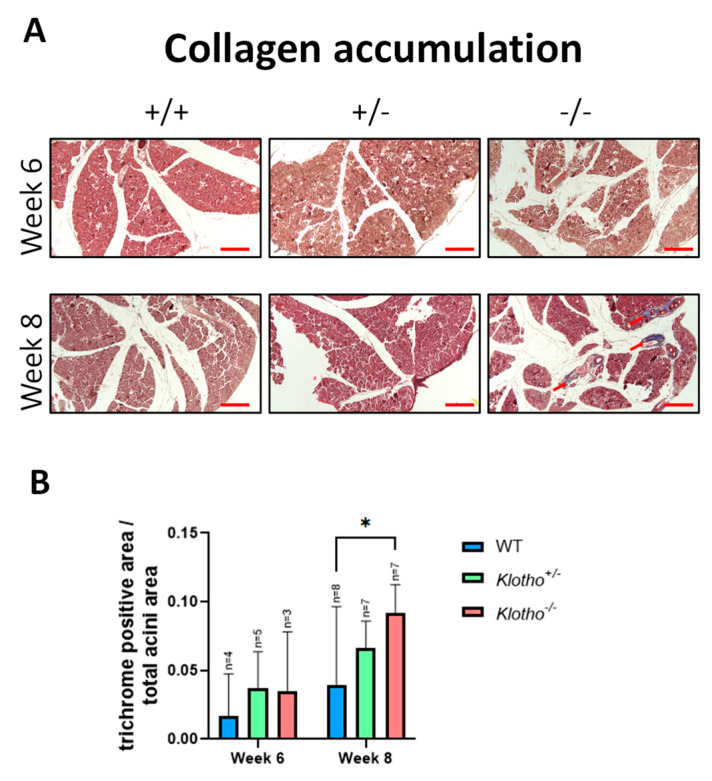
Trichrome staining for detection of collagen fiber deposition in the LGs and quantification analysis. (**A**) Representative photographs of LG trichrome staining of three genotypes at the 6th and 8th week of age. The Klotho^−/−^ LG at the 8th week of age showed evident collagen deposits, not found in the LGs of their littermates. Scale bars represent 100 μm. (**B**) Quantitative analysis, as presented by trichrome-positive area/total acini area, showed a significant increase in collagen fiber deposition in the Klotho^−/−^ LG compared to the Klotho^+/+^ LG, with * *p* < 0.05.

The LGs at the 6th and 8th week of age were stained with Masson’s trichrome and compared among the three genotypes. Evident deposition of collagen fibers was seen in most of the Klotho^−/−^ LGs at the 8th week of age, but not in those of the Klotho^+/+^ and Klotho^+/−^ genotypes at the same age (Figure 3A; indicated by red arrows). The quantitative analysis, as presented by the trichrome-positive area/total acini area, showed a significant increase in collagen fiber deposition in the Klotho^−/−^ LG compared to the Klotho^+/+^ LG (Figure 3B).

### 3.4. Increased Oxidative Stress

**Figure 4:**
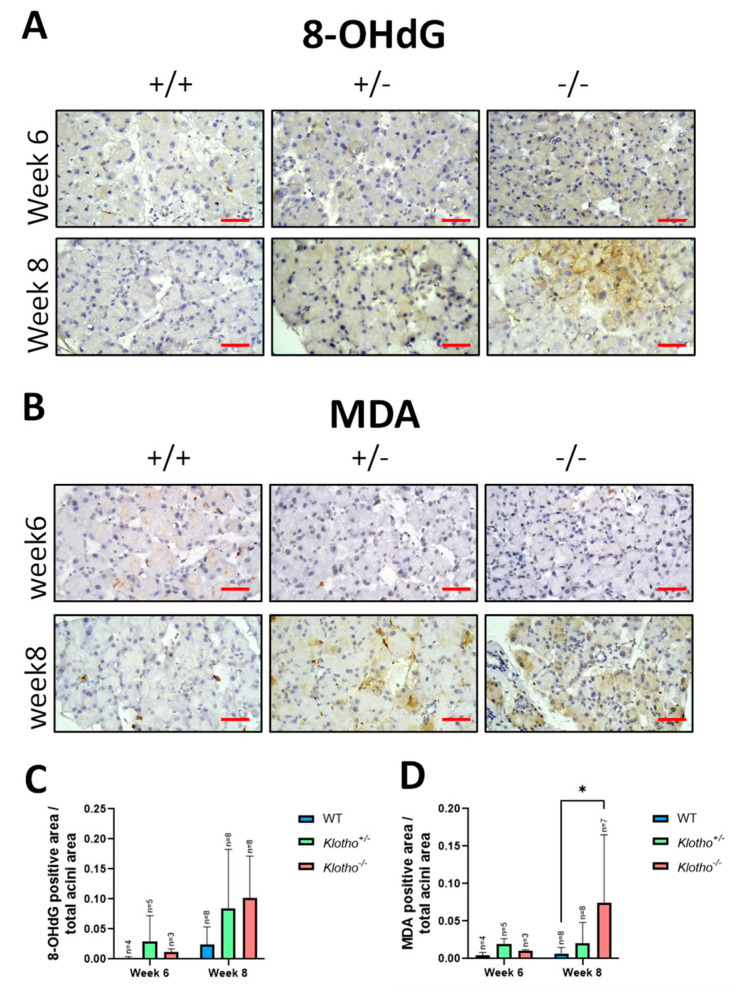
Representative photographs of LG immunohistochemical staining with 8-OHdG and MDA antibodies and quantification analysis. (**A**) Increased 8-OHdG signals were detected in the Klotho^+/−^ and Klotho^−/−^ LGs at the 8th week of age. (**B**) MDA signals were increased in Klotho^+/−^ and Klotho^−/−^ LGs at the 8th week of age. (**C**) Quantitative analysis of 8-OHdG as presented by 8-OHdG-positive area/total acini area. (**D**) Quantitative analysis of MDA as presented by MDA-positive area/total acini area. A significant increase in MDA expression was found in the Klotho^−/−^ LGs compared with the Klotho^+/+^ LGs at the 8th week of age, with * *p* < 0.05. The sample number of 8-OHdG and MDA was different due to missing data. Scale bars represent 25 μm.

Immunohistochemical staining was performed for 8-OHdG and MDA, markers of oxidative stress to DNA, and lipid peroxidation to further characterize the LG degeneration with the Klotho null mutation. For the 8-OHdG, no evident difference was found among the three genotypes at the 6th week of age (Figure 4A,C). At the 8th week of age, more 8-OHdG area over the total acini area was observed in the Klotho^+/−^ and Klotho^−/−^ LGs, although it was not statistically different compared to that in the Klotho^+/+^ LGs (Figure 4C). For the MDA, no signals were detected in the LGs of all three genotypes at the 6th week of age (Figure 4B,D). At the 8th week of age, more MDA expression, as presented by the MDA-positive area over the total acini area, was evident in both the Klotho^+/−^ and Klotho^−/−^ LGs, particularly in the Klotho^−/−^ LGs (Figure 4B), where a significant difference was found when compared with the Klotho^+/+^ LGs (Figure 4D).

### 3.5. Alteration of α-SMA Expression

**Figure 5:**
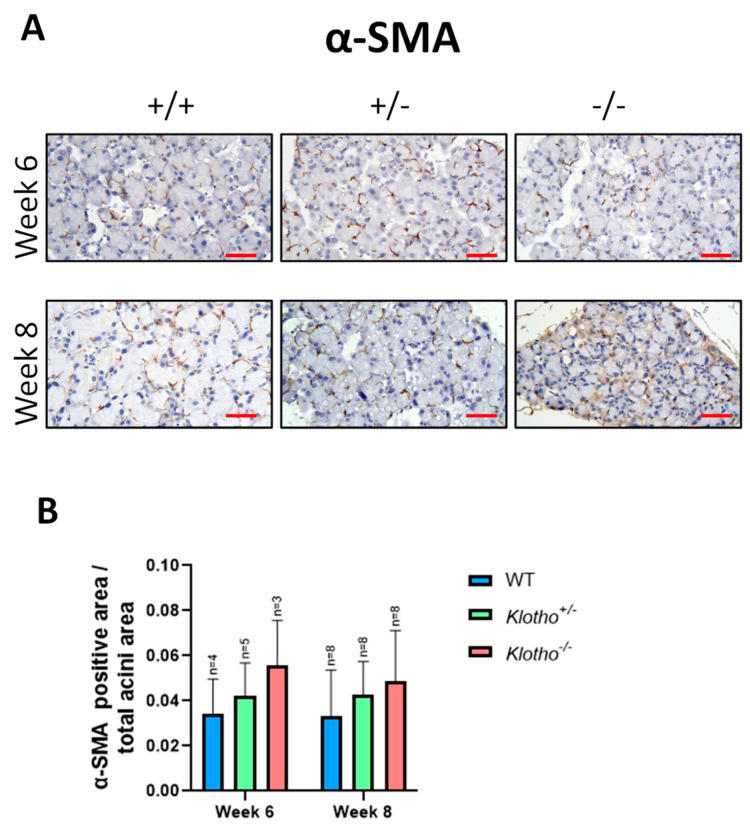
Immunohistochemical LG staining with α-SMA antibody and quantification analysis. (**A**) An evident increase in α-SMA signals surrounding the atrophic acini of the Klotho^−/−^ LG was detected at the 8th week of age. (**B**) Quantitative analysis, presented by α-SMA-positive area/total acini area, was found to increase in the Klotho^−/−^ LGs at both the 6th and the 8th week of age, as compared to those of Klotho^+/+^ and Klotho^+/−^ LGs, although without statistical significance. Scale bars represent 25 μm.

To examine whether the LG acinar atrophy was accompanied by extracellular matrix (ECM) remodeling, immunohistochemical staining using α-SMA (α-smooth muscle actin) antibody was performed in the LGs of all three genotypes at the 6th and 8th week of age. The expression of α-SMA was detected in all LGs (Figure 5A), with more signals found in the Klotho^−/−^ LGs at both the 6th and the 8th week of age when compared to the LGs in the Klotho^+/+^ and Klotho^+/−^ mice. Quantitative analysis, presented by α-SMA-positive area/total acini area, was found to increase in the Klotho^−/−^ LGs at both the 6th and the 8th week of age, compared to those of Klotho^+/+^ and Klotho^+/−^ LGs, although without statistical significance (Figure 5B).

### 3.6. Elevated MMP-2 and MMP-9 Expression

**Figure 6:**
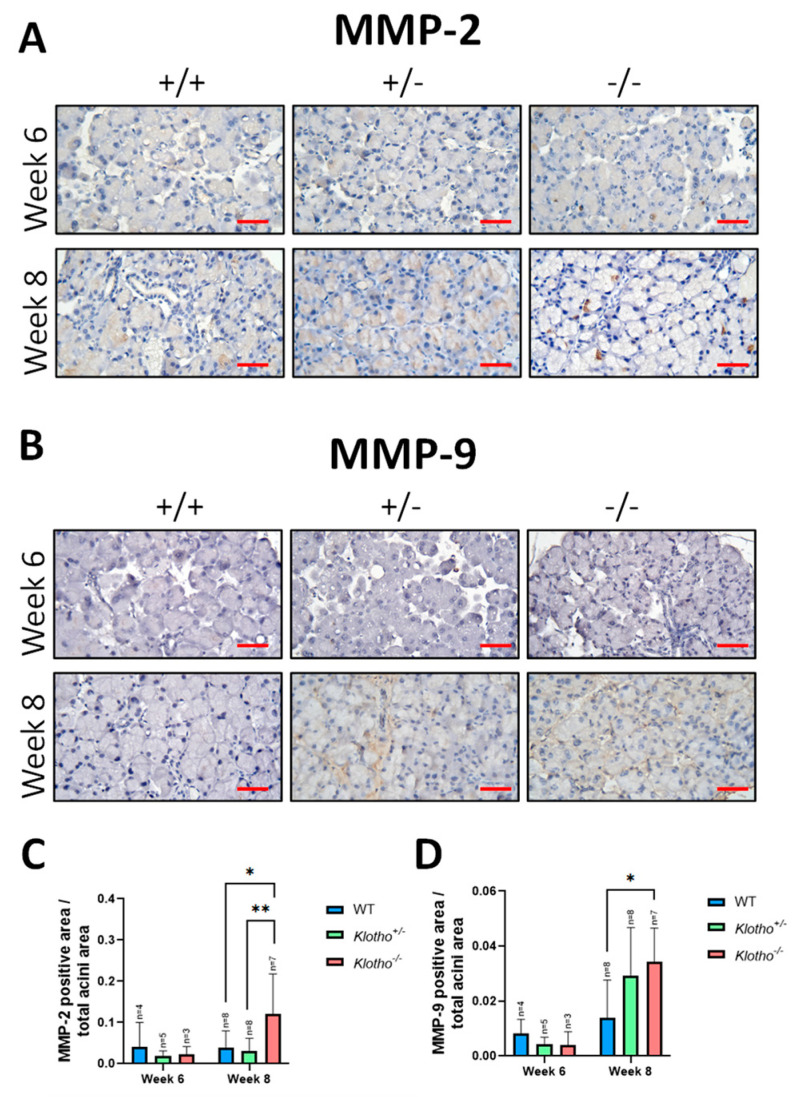
Representative photographs of LG immunohistochemical detection with MMP-2 and MMP-9 antibodies and quantification analysis. (**A**) No significant difference was observed for MMP-2 expression among all three genotypes at the 6th week of age. At the 8th week of age, a significant difference in MMP-2 expression was found between the Klotho^+/+^ and the Klotho^−/−^ LGs and between the Klotho^+/−^ and the Klotho^−/−^ LGs. (**B**) For MMP-9 expression, a significant difference was observed between the Klotho^+/+^ and the Klotho^−/−^ LGs, but not between the Klotho^+/+^ and the Klotho^−/−^ LGs at the 8th week of age. (**C**) Quantification of MMP-2 expression, presented by MMP-2-positive area/total acini area, showed a significant increase in the Klotho^−/−^ LG compared with the Klotho^+/+^ and the Klotho^+/−^ LGs at the 8th week of age, with * *p* < 0.05 and ** *p* < 0.01, respectively. (**D**) Quantification of MMP-9 expression at the 8th week of age, presented by MMP-9-positive area/total acini area, showed a significant increase in the Klotho^−/−^ LG compared with the Klotho^+/+^ LG, with * *p* < 0.05, but not with the Klotho^+/−^ LG. Scale bars represent 25 μm.

To further elucidate the ECM remodeling activities within LGs with the Klotho null mutation, immunohistochemical staining was performed for MMP-2 (matrix metalloproteinase-2) and MMP-9 (matrix metalloproteinase-9) expression in the three genotypes at both the 6th and the 8th week of age. At the 6th week of age, no significant difference was observed for MMP-2 (Figure 6A,C) and MMP-9 (Figure 6B,D) among the three genotypes. At the 8th week of age, a significant elevation in MMP-2 expression was found between the Klotho^+/+^ and the Klotho^−/−^ LGs and also between the Klotho^+/−^ and the Klotho^−/−^ LGs (Figure 6A,C). For MMP-9 expression, a significant increase was observed between the Klotho^+/+^ and the Klotho^−/−^ LGs, but not between the Klotho^+/+^ and the Klotho^+/−^ LGs at the 8th week of age (Figure 6B,D).

### 3.7. Increased Expression of Tyrosine Hydroxylase and Vasoactive Intestinal Polypeptide

**Figure 7:**
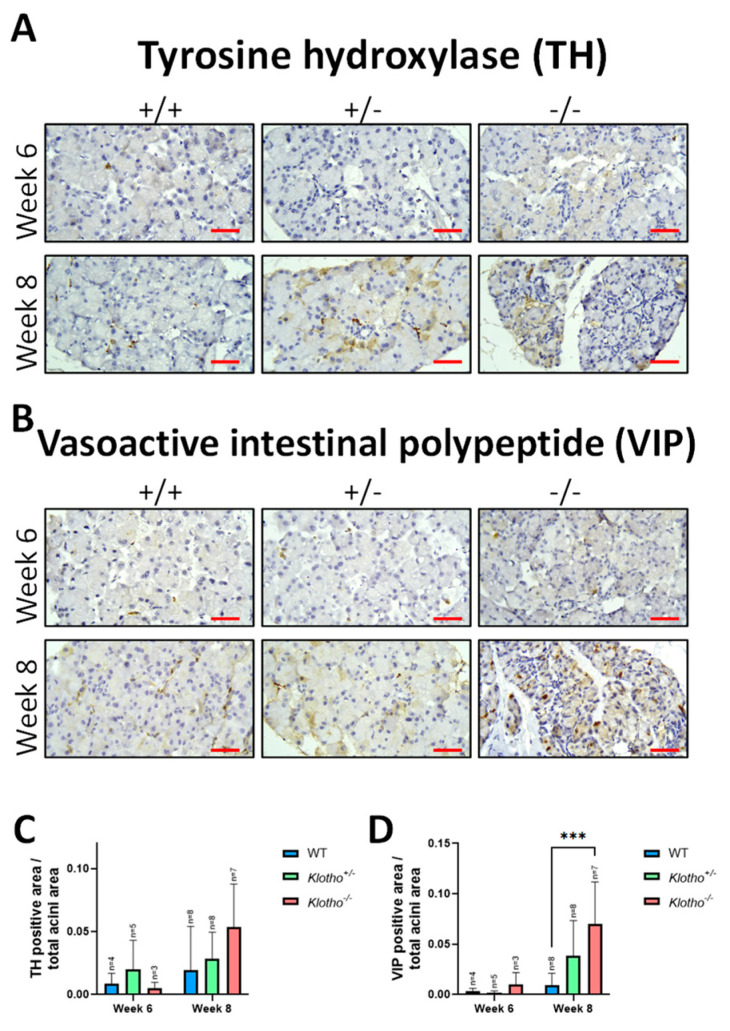
Immunohistochemical stains with tyrosine hydroxylase (TH) and vasoactive intestinal polypeptide (VIP) antibodies. (**A**) TH signals were increased in the Klotho^+/−^ and the Klotho^−/−^ lacrimal glands at the 8th week of age. (**B**) VIP signals were elevated in the Klotho^−/−^ LGs at the 6th and 8th weeks of age. (**C**) Quantification analysis of TH expression, presented by TH-positive area/total acini area, showed an increase in the Klotho^−/−^ LGs, compared with the Klotho^+/+^ LGs, although without statistical difference. (**D**) Quantification of VIP expression, presented by VIP-positive area/total acini area, showed a significantly elevated expression in the Klotho^−/−^ LGs, compared with the Klotho^+/+^ LGs, with *** *p* < 0.001. Scale bars represent 25 μm.

The LG’s sympathetic and parasympathetic nerve transductions were examined via the immunohistochemical detection of tyrosine hydroxylase (TH) and VIP (vasoactive intestinal polypeptide). No significant difference in TH expression was found among the three genotypes at the 6th week of age (Figure 7A,C). At the 8th week of age, increased TH expression was found, particularly in the Klotho^−/−^ LGs, compared with the Klotho^+/+^ LGs, although without statistical difference. For the VIP expression, increased expression was seen in the Klotho^−/−^ LGs in the 6th week, and this increase was maintained in the 8th week and was highly significant when compared with that of the Klotho^+/+^ LGs (Figure 7B,D).

### 3.8. Decreased FGF-23 Expression

**Figure 8:**
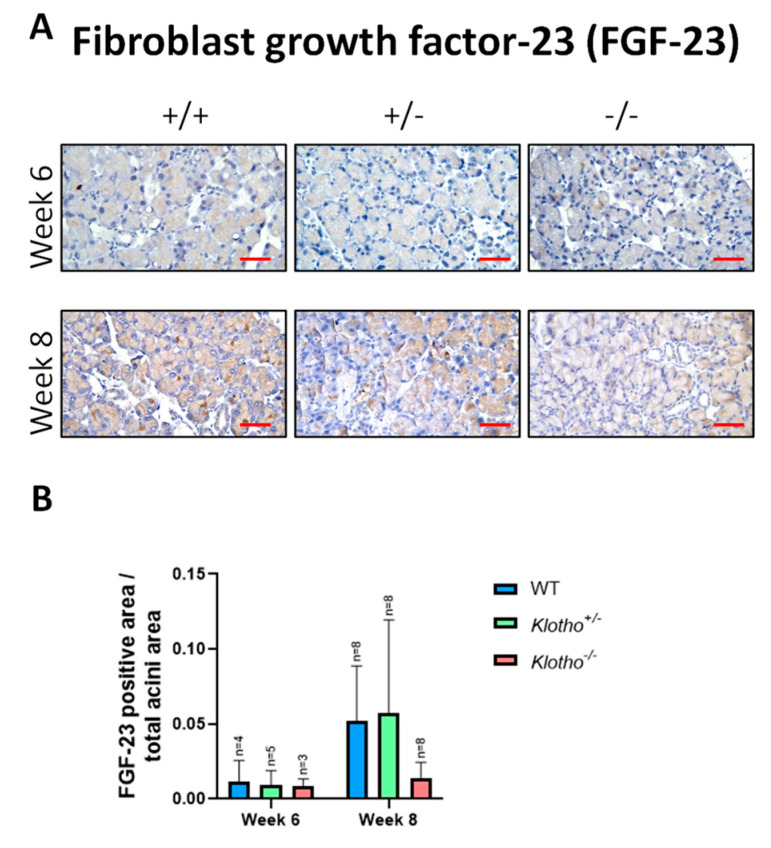
Representative photographs of LG immunohistochemical staining with FGF-23 antibody and quantitative analysis. (**A**) FGF-23 expression was decreased in the Klotho^−/−^ LGs, as compared to those in the Klotho^+/−^ and the Klotho^+/+^ LGs at the 8th week of age. (**B**) Quantitative analysis, presented by FGF-23-positive area/total acini area, showed decreased FGF-23 protein expression with the Klotho null mutation, although without statistical difference. Scale bars represent 25 μm.

Since we had found a lack of membrane-bound Klotho protein in the Klotho^+/+^ LGs (Figure 1E), the Klotho-FGF-23 complex would presumably form only through the binding of the secreted form of the Klotho protein from blood circulation. However, whether the LG’s FGF-23 expression could be maintained with the Klotho null mutation remained undetermined. The results of immunohistochemical detection showed that FGF-23 expression was decreased in the Klotho^−/−^ LGs, as compared to those in the Klotho^+/−^ and the Klotho^+/+^ LGs at the 8th week of age (Figure 8A), although without significant differences (Figure 8B).

## 4. Discussion

The Klotho mutation has been found to lead to age-related atrophy in the skin, genital organs, and thymus [11]. Nevertheless, no detailed description of lacrimal gland atrophy related to the Klotho mutation had been reported in the literature. The present study examined whether LG aging can be found with the Klotho null mutation. The results showed general characteristics of LG aging, including atrophy, thickened capsule, collagen deposition, and reduced tear volume, mostly found at the 8th week of age. LG atrophy has been shown to indicate LG malfunction and a reduction in tear secretion, which will eventually lead to a dry eye status [6,14,15]. Accordingly, our results of reduced tear secretion reflected the lacrimal gland atrophy in the Klotho^−/−^ mice, in contrast to the Klotho^+/+^ and the Klotho^+/−^ mice.

The underlying mechanism for the LG atrophy and functional loss in the Klotho^−/−^ mice can be multi-fold. Firstly, the elevation of chronic oxidative stress is a substantial and widely acclaimed cause of aging [16,17]. Oxidative stress is also implicated in dry eye pathogenesis in the aged population [18]. MDA has been used as a marker for lipid peroxidation in the LGs [19,20], while 8-OHdG often represents the extent of DNA damage during aging [1,10,16]. In the present study, we found that MDA and 8-OHdG were increased in the LGs with the Klotho null mutation at the 8th week of age, although a significant difference was only found for the MDA. This status of chronic elevation of oxidative stress is likely to lead to a vicious cycle of oxidative stress−inflammation [21,22], which will eventually trigger LG atrophy.

Secondly, to further elucidate the LG atrophy with the Klotho null mutation, we examined the changes in MMP-2 and MMP-9, since chronic inflammation is known to lead to an increase in matrix metalloproteinases (MMPs) in the LG, especially MMP-2 and MMP-9, and cause extracellular matrix (ECM) remodeling [23]. Our results showed increased MMP-2 and MMP-9 expression in the Klotho mutant LGs. The consequence of this increase would be constant ECM remodeling and the disruption of the LG’s acinar structure and repair, as indicated by many lines of evidence that have been previously reported [23,24,25]. In addition, the constant ECM remodeling would thicken the connective tissue sheath [26], also found in the Klotho^−/−^ LG capsules at the 8th week of age in the present study. Intriguingly, MMP-2 and MMP-9 are collagen degradation enzymes [23,25]. The increase in both enzymes would lead to collagen degradation, contrary to collagen fiber deposition shown by trichrome staining in the present study. One potential explanation for the collagen deposition in the Klotho^−/−^ LGs may be due to the differential digestive activities of both enzymes. MMP-2 can cleave gelatin and type I and IV collagens, while MMP-9 mainly digests type IV collagen and cannot directly undertake the proteolysis of collagen I [23,25]. Collagen deposition may be type-specific, depending on the local imbalance of MMP-2 and MMP-9 distribution, which should be further investigated.

Thirdly, LG atrophy with the Klotho null mutation was also indicated by another line of evidence showing the changes in myoepithelial cells. α- SMA is the major marker of myoepithelial cells [27,28]. In normal LGs, myoepithelial cells surrounding the acini are maintained [27,29,30]. During normal aging, fewer myoepithelial cells surrounding glands are found, as represented by lower α- SMA marker expression [31,32]. Interestingly, our results showed increased α- SMA signals in the LGs with the Klotho null mutation at the 8th week of age. Notably, however, α-SMA has also been known as a reliable marker to characterize the mesenchymal products generated by the epithelial–mesenchymal transitions (EMTs) that occur during the development of fibrosis in various organs [33]. EMTs and their reverse processes, METs (mesenchymal–epithelial transitions), allow the cells to switch from an epithelial state to a mesenchymal one and vice versa [33]. Through a homeostatic balance between EMTs and METs, the LG is highly regenerative and can repair itself even after substantial damage. However, chronic oxidative stress and inflammation may disrupt the homeostatic balance between EMTs and METs, favoring EMTs instead. In the present study, the accelerated aging with the Klotho null mutation was found to lead to the increase in α-SMA, MMP-2, and MMP-9 and the deposition of collagen in the LG, which may be regarded as a scenario of EMT and its related extracellular matrix remodeling that results in the failure of LG repair and subsequent LG atrophy.

Fourthly, the sensory nerve endings on the ocular surface transmit signaling to the brain, and the latter controls the tear secretion through signals toward the LG. The secretion of aqueous, electrolyte, and protein contents from the LG to the ocular surface is regulated by the dual effects of both sympathetic and parasympathetic nerves [19,20]. Through this neural regulation circuit, the ocular surface can be kept under a homeostatic status and moisture is maintained [20]. Tyrosine hydroxylase (TH) and vasoactive intestinal polypeptide (VIP) may represent the activities under sympathetic and parasympathetic regulation, respectively. In the present study, we found that both TH and VIP were increased in the Klotho^−/−^ LGs, which would stimulate tear secretion, contrary to the finding that the tear volume was decreased. This contradictory finding also depicted the failure of LG repair, since a regular repair would accept proliferating and differentiating epithelial cells and nerves from adjacent uninjured tissue to move to the wound site [24]. With the Klotho null mutation, if increased α-SMA expression favors being the mesenchymal products generated by the EMTs, those cells containing α-SMA would lose their myoepithelial cell identity, resulting in diminishing roles in the maintenance of the LG structure and the repair of epithelial cells and nerves. Thus, the increased signals of TH and VIP in the Klotho^−/−^ LGs would be more like a compensatory effect than an effective nerve activation to trigger tear secretion in the Klotho null LGs.

Fifthly, for normal LG gland development and epithelium repair, the binding of growth factors (for example, fibroblast growth factors, FGFs) to heparan sulfate creates morphogenetic gradients to control epithelial polarity and direct LG epithelial growth and migration [27]. The secretory form of the Klotho protein acts as a circulating co-receptor for FGF-23, promoting interaction between FGF-23 and membrane-bound FGF receptors (FGFRs) [11]. This interaction facilitates the binding of FGF-23 to cell types that do not express Klotho and mediate temporary responsiveness to FGF-23 [34]. Our results in the present study showed that Klotho^−/−^ LGs expressed less FGF-23 than their wildtype and heterozygous counterparts, likely due to a lack of normal Klotho protein to function as the co-receptor. Thus, the Klotho^−/−^ mice would be at least partly deprived of the FGF-23 involvement in LG development and epithelium repair, resulting in a smaller LG size and LG atrophy. In addition, since no Klotho protein was present in the normal LGs, all the characteristics of LG aging with the Klotho null mutation must be mediated indirectly, presumably due to the failure of phosphate excretion and subsequent adverse effects.

Klotho mutant mice have been extensively used as a study model for aging in various organs. Still, no information regarding the effects of the Klotho mutation on LG structure and function has been elucidated. Currently, the most commonly used mouse dry eye models are induced through various methods, including environmental desiccation, lacrimal gland extraction, botulinum toxin injection, and chemical or physical damage by using benzalkonium chloride (BAC) eye drops or UVB irradiation [3,20,35,36]. However, tear secretion and the maintenance of ocular surface moisture is under multiple regulations through a network of apparatus and involves LG, the meibomian gland, and conjunctival goblet cells [37]. The long-term effects of aging on the degeneration of the aforementioned apparatus are most likely responsible for dry eye in humans. No study model of gradual lacrimal atrophy for dry eye disease has existed. Since our data showed that the Klotho mutation leads to a gradual process of LG degeneration from normal status to pathogenesis, the Klotho mutant mouse model appears more suitable for studying dry eye as a human disease model. Another advantage of Klotho mutants is the accelerated pathogenesis, which is unavailable from the normal aging models. It takes more than one year for normal mice to exhibit LG atrophy [3,38] and capsular thickening [26,39]. In contrast, our data showed that the Klotho^−/−^ LGs took only 8 weeks to show these age-related pathological characteristics. This accelerated pathogenesis would facilitate studies on LG aging and its related dry eye diseases.

## 5. Conclusions

Here, we document the morphological and pathological changes in the mouse Klotho^−/−^ LGs. The findings include reduced LG size and tear secretion, thickened LG capsule, acinar atrophy, elevated oxidative stress, collagen fiber deposition, and presumably the loss of myoepithelial cell identity through the process of epithelial–mesenchymal transition, as well as the compensatory upregulation of LG neural activities. This scenario would impede epithelial repair and lead to LG atrophy, resulting in dry eye status. All these events are representative of the characteristics of human LG aging. With the advantages of accelerated aging and a congenital nature, the Klotho null mutant mouse may serve as a model for age-related lacrimal gland degeneration and congenital dry eye disease.

## Figures and Tables

**Table 1:** The PCR primers and genotypes indicated by the PCR product lengths. The same set of primers was used to detect either heterozygous or homozygous genotypes.

	Primer Sequence	PCR Product Length	Genotype
Forward	5′-GATGGGGTCGACGTCA-3′	186 bp only	Wildtype (^+/+^)
Reverse	5′-TAAAGGAGGAAAGCCATTGTC-3′		
Forward	5′-GCAGCGCATCGCCTTCTATC-3′	186 bp and 455 bp	Heterozygous (^+/−^)
Reverse	5′-ATGCTCCAGACATTCTCAGC-3′		
Forward	5′-GCAGCGCATCGCCTTCTATC-3′	455 bp only	Homozygous (^−/−^)
Reverse	5′-ATGCTCCAGACATTCTCAGC-3′		

## Data Availability

The data presented in this study are available from the corresponding author upon reasonable request.
